# Evaluation of Gene Expression Data From Cybrids and Tumours Highlights Elevated *NDRG1*-Driven Proliferation in Triple-Negative Breast Cancer

**DOI:** 10.1177/1178223420934447

**Published:** 2020-06-22

**Authors:** Akanksha Mishra, Maria Bonello, Adam Byron, Simon P Langdon, Andrew H Sims

**Affiliations:** 1 Applied Bioinformatics of Cancer, Cancer Research UK Edinburgh Centre, MRC Institute of Genetics & Molecular Medicine, The University of Edinburgh, Edinburgh, UK; 2 Division of Pathology, The University of Edinburgh, Edinburgh, UK; 3 Cancer Research UK Edinburgh Centre, MRC Institute of Genetics & Molecular Medicine, The University of Edinburgh, Edinburgh, UK

**Keywords:** NDRG1, breast cancer, cybrids

## Abstract

**Background:**

Triple-negative breast cancer is an aggressive type of breast cancer with high risk of recurrence. It is still poorly understood and lacks any targeted therapy, which makes it difficult to treat. Thus, it is important to understand the underlying mechanisms and pathways that are dysregulated in triple-negative breast cancer.

**Methods:**

To investigate the role of mitochondria in triple-negative breast cancer progression, we analysed previously reported gene expression data from triple-negative breast cancer cybrids with SUM-159 as the nuclear donor cell and SUM-159 or A1N4 (c-SUM-159, c-A1N4) as the mitochondrial donor cells and with 143B as the nuclear donor cell and MCF-10A or MDA-MB-231 (c-MCF-10A, c-MDA-MB-231) as the mitochondrial donor cells. The role of potential biomarkers in cell proliferation and migration was examined in SUM-159 and MDA-MB-231 cells using sulforhodamine B and wound healing assays.

**Results:**

Rank product analysis of cybrid gene expression data identified 149 genes which were significantly up-regulated in the cybrids with mitochondria from the cancer cell line. Analysis of previously reported breast tumour gene expression datasets confirmed 9 of the 149 genes were amplified, up-regulated, or down-regulated in more than 10% of the patients. The genes included *NDRG1, PVT1*, and *EXT1*, which are co-located in cytoband 8q24, which is frequently amplified in breast cancer. *NDRG1* showed the largest down-regulation in the cybrids with benign mitochondria and was associated with poor prognosis in a breast cancer clinical dataset. Knockdown of *NDRG1* expression significantly decreased proliferation of SUM-159 triple-negative breast cancer cells.

**Conclusions:**

These results indicate that mitochondria-regulated nuclear gene expression helps breast cancer cells survive and proliferate, consistent with previous work focusing on an Src gene signature which is mitochondria regulated and drives malignancy in breast cancer cybrids. This is the first study to show that mitochondria in triple-negative breast cancer mediate significant up-regulation of a number of genes, and silencing of *NDRG1* leads to significant reduction in proliferation.

## Introduction

Triple-negative breast cancer is characterised by absence of oestrogen receptor, progesterone receptor, and HER2 receptor amplification. Its pathogenesis is still not well understood, and it is associated with poor prognosis and high recurrence rate. Therefore, it is important to improve our understanding of the pathophysiology of triple-negative breast cancers in order that better therapeutic targets can be identified.^1^ Previous studies have reported the role of mitochondria-mediated metabolic reprogramming to fatty acid β-oxidation in triple-negative breast cancer.^2^ Mitochondria in the cancer cell respond to the change in nutrient status by sending signals in the form of metabolites, reactive oxygen species (ROS), or changes in adenosine diphosphate: adenosine triphosphate (ADP: ATP) ratio, which affects both nuclear gene expression and cytosolic signalling pathways.^1,3^ These mitochondria-mediated changes in nuclear gene expression help cancer cells survive and proliferate. Trans-mitochondrial cybrids are an important tool to study the role of mitochondria in a defined nuclear background. Online portals such as the Gene Expression Omnibus (GEO) enable researchers to access multiple sets of gene expression data, which can be analysed and visualised using platforms such as cBioPortal for The Cancer Genome Atlas (TCGA) cancer genomics data.^4,5^ Integration of such analyses with laboratory-based studies represents a powerful approach to understand cancer biology phenotypes more deeply, providing greater opportunity for mechanistic insights in the system under study.

This study considered changes in nuclear gene expression when benign mitochondria are introduced into a metastatic nuclear background, using microarray data^2,6^ from triple-negative breast cancer cybrids to identify potential novel targets. Publicly available gene expression datasets of breast tumours were used to prioritise potential candidates, which were validated using short interfering RNA (siRNA)-mediated silencing to demonstrate the effectiveness of the approach. Among 9 genes which were significantly down-regulated, *NDRG1* was most affected and was also confirmed to be amplified and up-regulated in a significant proportion of triple-negative breast cancer patients.

## Material and Methods

### Cell culture

The human triple-negative breast cancer cell lines MDA-MB-231 and SUM-159, purchased from American Type Culture Collection (ATCC) were cultured in Dulbecco’s modified Eagle’s medium (DMEM; Sigma-Aldrich, USA) supplemented with 10% foetal calf serum (FCS), 50 U mL^−1^ penicillin, and 50 mg mL^−1^ streptomycin (all Sigma-Aldrich, USA). Cells were maintained at 5% CO_2_ at 37°C in a humidified incubator.

### Reagents and antibodies

Phosphatase inhibitor cocktail 2, phosphatase inhibitor cocktail 3, cOmplete protease inhibitor cocktail, and aprotinin were obtained from Sigma-Aldrich. *NDRG1* siRNA was Dharmacon SMARTpool ON-TARGETplus (L-010563-00-0005, https://horizondiscovery.com/products/gene-modulation/knockdown-reagents/sirna/PIFs/ON-TARGETplus-siRNA-Reagents-Human?nodeid=entrezgene-10397), the ON-TARGETplus non-targeting siRNA (D-001810-01-05) and DharmaFECT 4 transfection reagent (T-2004-02) were from Horizon Discovery. Anti-NDRG1 (D8G9) XP rabbit antibody (9485S) and pre-stained protein markers (13953S) were obtained from Cell Signalling Technology.

### Short interfering RNA transfection

The MDA-MB-231 cells and SUM-159 cells were seeded at a density of 3.00 × 10^5^ and 2.75 × 10^5^ cells, respectively, in a 6-well tissue culture plate. After 24 hours, cells were transfected with 25 nM of *NDRG1*-targeting siRNA or non-targeting siRNA using DharmaFECT 4. Culture medium was changed to DMEM + 10% FCS + AB medium 24 hours after treatment. Cells were lysed at 48, 72, 96, and 120 hours after siRNA treatment.

### Cell lysis

Tissue culture plates were washed with phosphate-buffered saline (PBS) and treated with lysis buffer (50 mM Tris-HCl, pH 7.5, 5 mM EGTA, pH 8.5, and 150 mM NaCl) containing 1% Triton X-100, 100 μL phosphatase inhibitor cocktail 2, 100 μL phosphatase inhibitor cocktail 3, 50 μL aprotinin and 1× cOmplete protease inhibitor cocktail. Lysates were clarified by centrifugation at 13 000 × *g* for 5 minutes. All procedures were performed at 4°C. Protein concentration was determined using a bicinchoninic acid (BCA) assay, and cell lysates were stored at –80°C.

### Western blot analysis

Samples containing 40 μg of protein were resolved by sodium dodecyl sulphate–polyacrylamide gel electrophoresis. Resolved proteins were transferred to polyvinylidene fluoride membrane, which was then blocked using a 50:50 solution of PBS and Odyssey Blocking Buffer (LI-COR Biosciences, USA). The membrane was then incubated with anti-NDRG1 antibody (1:1000) overnight at 4°C. The membrane was washed and incubated with secondary antibody (1:10 000) and imaged using an Odyssey infrared imager (LI-COR Biosciences, USA). Anti-α-tubulin antibody (Abcam) diluted 1:8000 was used as a loading control.

### Immunocytochemistry

The MDA-MB-231 and SUM-159 cells were transfected with *NDRG1*-targeting siRNA. Cells were washed with PBS and fixed in acetone for 10 minutes at 4°C. Fixed cells were washed twice in PBS containing 0.1% Tween 20 (PBS-T) and placed in H_2_O_2_ solution for 10 minutes. Cells were washed twice with PBS-T and placed in DAKO blocking solution for 10 minutes at room temperature. Primary antibody diluted 1:800 as recommended in DAKO antibody dilution solution was added for 1 hour at room temperature. Cells were washed with PBS-T and incubated with appropriate secondary antibody (Envision labelled polymer) for 30 minutes at room temperature. Cells were treated with DAB solution for 10 minutes, followed by washing and counterstaining with haematoxylin for 40 seconds. After repeated cycle of washes with alcohol and xylene, cells were dried and observed by light microscopy.

### Cell proliferation assay

Cells were seeded in triplicate in 96-well plates (800 cells per well). Cells were treated with 25 nM *NDRG1* siRNA after 24 hours. Following siRNA treatment, cells were fixed at 48, 72, 96, and 120 hours using 50 µL 25% trichloroacetic acid for 1 hour at 4°C. Plates were washed with water and left to dry. Next, 50 µL sulforhodamine B (SRB) dye were added, and plates were incubated for 30 minutes at room temperature. Plates were washed with 1% glacial acetic acid solution and left for drying. Once dry, 150 µL Tris buffer was added, and after 1 hour, plates were read at optical density 540 nm using a BP800 Microplate Reader (BIOHIT Healthcare, Finland).

### Wound healing (scratch) assay

The SUM-159 and MDA-MB-231 cells were seeded at 275 000 and 300 000 cells per well, respectively, in a 6-well tissue culture plate. The cells were treated with *NDRG1* siRNA after 24 hours, and cell culture medium was changed 24 hours post-transfection. A scratch was made using a 200-µL pipette tip at 48 and 72 hours post-transfection for SUM-159 and MDA-MB-231 cells, respectively. Photomicrographs were taken using a light microscope at 0, 6, and 24 hours using a 2.5× objective.

### Acquisition of gene expression data

Gene expression data of transmitochondrial cybrids, with moderately metastatic triple-negative breast cancer cells SUM-159 as the common nuclear background, and mitochondria from benign breast epithelium (A1N4) or moderately metastatic triple-negative breast cancer cells (SUM-159), were downloaded from the NCBI GEO repository (GSE72319).^2^ Another dataset of transmitochondrial cybrid data, with metastatic osteosarcoma-derived 143B ells as the common nuclear background, and mitochondria from noncancerous mammary epithelial MCF-10A cells or breast cancer MDA-MB-468 cells, was gratefully obtained on request from Benny Kaipparettu (Baylor College of Medicine, Houston, Texas, USA).^6^

### Data processing and analysis

Bioconductor^7^ packages from the R programming language^8^ were used for analysis. A custom chip definition file (CDF) from brainarray^9^ was used to map Affymetrix probe level data to Entrez gene ID, and robust multi-array averaging implemented by the *affy* package was used for normalisation. The dataset was filtered using mas5calls implemented in the *affy* package to remove genes which were absent in more than 4 samples (out of a total of 6 samples). The Rank Products method^10^ of analysis was implemented by the *rankprod* package to identify differentially expressed genes. Pathway analysis was performed using the *SPIA* package (R/Bioconductor). Gprofiler was used to perform gene ontology enrichment analysis. cBioPortal (www.cbioportal.org/) was used to examine the genes in publicly available breast tumour datasets. Survival analysis was performed with the survivALL package.^11^

## Results

### Cybrid-specific gene expression is associated with cell proliferation, blood vessel morphogenesis, response to hypoxia, and focal adhesions

**Figure 1.**
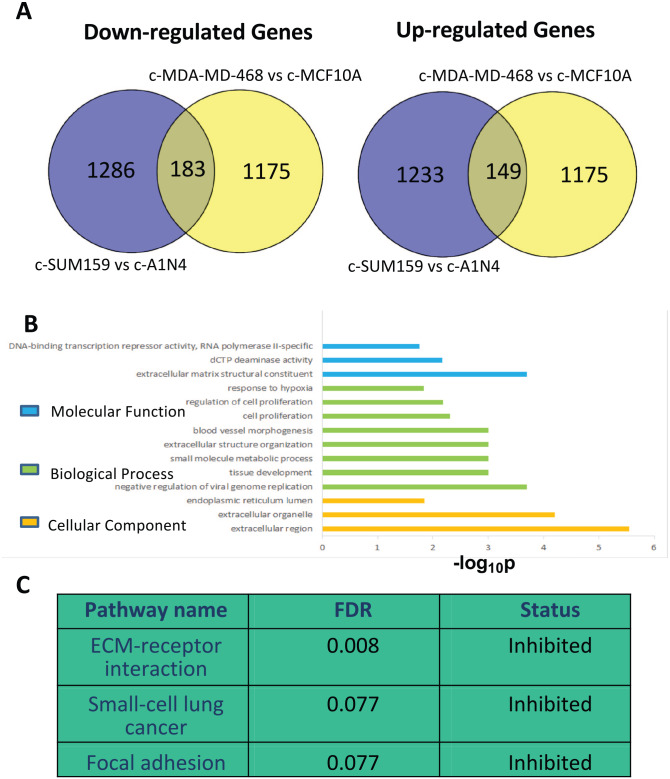
Analysis of cybrid-specific gene expression in triple-negative breast cancer cells. (A) Venn diagrams showing the 183 genes down-regulated and 149 genes up-regulated in both SUM-159 and 143B triple-negative breast cancer cybrid models. (B) Gene ontology enrichment analysis of the 149 genes significantly up-regulated in both triple-negative breast cancer cybrid models. (C) Signalling pathway impact analysis showing 3 significantly inhibited pathways (false discovery rate, FDR < 0.1).

To identify genes whose expression is regulated by mitochondria derived from normal and malignant breast cancer cell line against the background of malignant breast cancer cell line, we compared gene expression data derived from the cancer and normal cybrids (c-A1N4 vs c-SUM-159 and c-MCF-10A vs c-MDA-MB-231). Rank Product analysis identified 2851 and 2682 genes (*P* < .05) differentially expressed in the respective datasets, with similar proportions up- and down-regulated. A total of 622 genes were differentially expressed across the datasets, of which 149 genes were up-regulated and 183 genes were down-regulated in cybrids with mitochondria from cancer cells compared with cybrids from benign cells (Figure 1A). Gprofiler was used to examine the over-representation of gene ontology terms for the 149 genes up-regulated in both SUM-159 and 143B triple-negative breast cancer cybrid models. These were found to be enriched in biological processes such as cell proliferation, blood vessel morphogenesis, and response to hypoxia (Figure 1B). Signalling pathway impact analysis identified 7 pathways enriched among up-regulated genes, including ECM-receptor interaction, focal adhesion, and small cell lung cancer pathways (Figure 1C).

### Cybrid-specific genes are differentially expressed in triple-negative breast tumours

**Figure 2.**
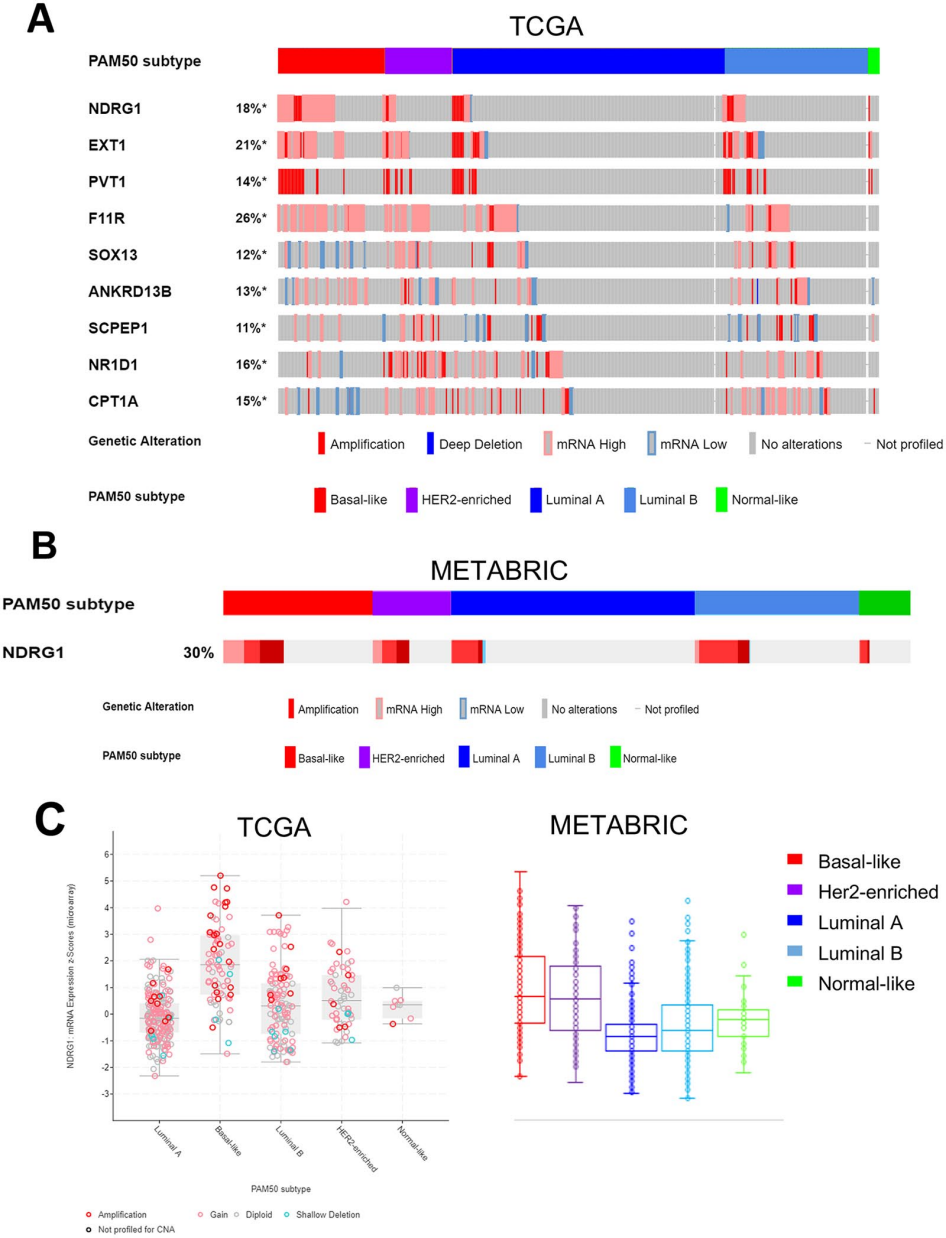
Cybrid-specific potential targets are differentially expressed in breast tumours. (A) Dysregulated genes in more than 10% of the patients in the TCGA breast cancer dataset,^12^ of which 3 genes, *NDRG1, EXT1*, and *PVT1*, located in the frequently amplified 8q24 cytoband. (B) *NDRG1* was also amplified or overexpressed in 30% of the METABRIC dataset.^13^ (C) The highest levels of *NDRG1* expression are observed in the basal subtype in both datasets. TCGA indicates The Cancer Genome Atlas.

To investigate the relevance of the expression of the cybrid-associated genes, we examined gene expression and copy number variation in publicly available gene expression datasets. Out of the 149 genes, 9 were observed to be amplified, up-regulated, or down-regulated in more than 10% of the 963 patients in the TCGA breast cancer dataset.^12^ Of these, 3 genes, *NDRG1, EXT1*, and *PVT1*, are known to be located in the frequently amplified 8q24 cytoband in breast cancer (Figure 2A). *NDRG1* had the greatest fold change in cybrids and was also observed to be amplified and overexpressed in a substantial proportion of breast cancer patient samples. *NDRG1* was up-regulated in cybrids with cancer cell–derived mitochondria (2.7-fold change in c-SUM-159 vs c-A1N4).^13^ Comparing *NDRG1* gene expression across breast cancer subtypes, it was observed that median expression of *NDRG1* was much greater in the basal triple-negative subtype (Figure 2B and C) in both the TCGA and METABRIC^13^ datasets (Figure 2B and C).

### Silencing of NDRG1 reduces proliferation, but does not affect migration of breast cancer cells

**Figure 3.**
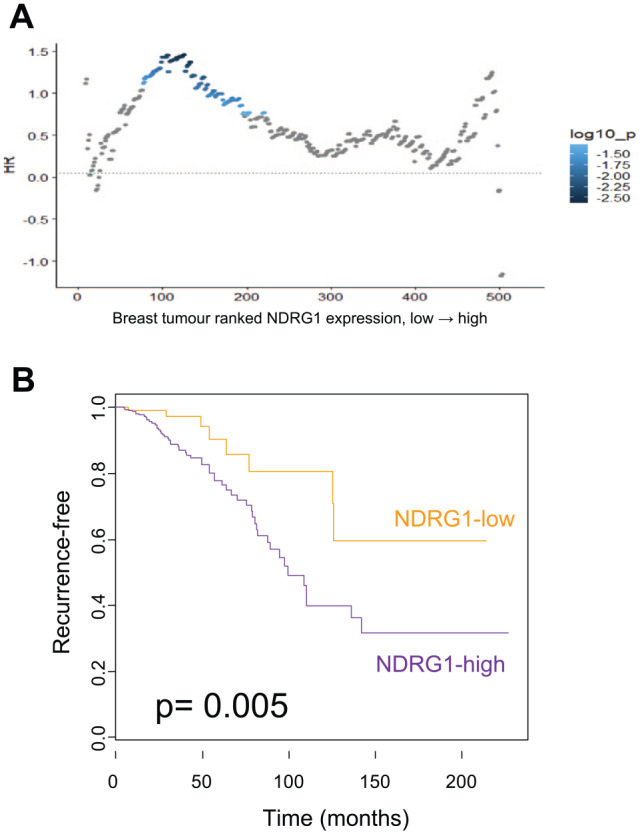
Elevated *NDRG1* expression is associated with poor prognosis in patients with breast cancer. (A) Comprehensive survival analysis using survivALL demonstrates that high levels of *NDRG1* expression are associated with poor outcome in the TCGA dataset. Over 25% of all possible cut-points are significant (*P* < .05), highlighted in shades of blue, lower = darker. (B) Kaplan-Meier plot showing the most significant cut-point where high *NDRG1* expression is associated with worse outcome. TCGA indicates The Cancer Genome Atlas.

**Figure 4.**
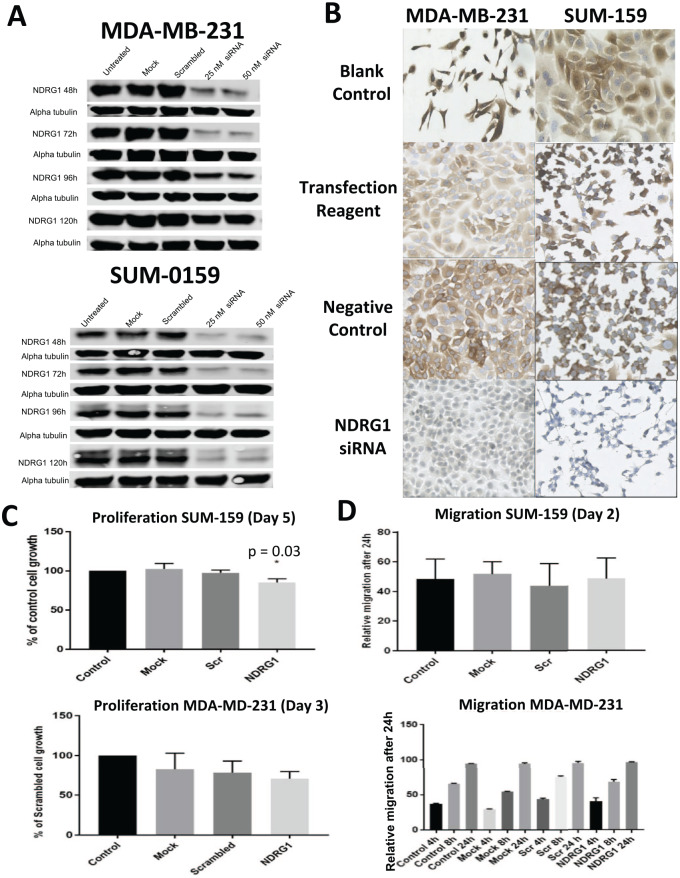
Silencing *NDRG1* in SUM-159 and MDA-MB-231 cells. (A) Western blot analysis of lysates from MDA-MB-231 and SUM-159 cell lines transfected with *NDRG1* siRNA at 48, 72, 96, and 120 hours post-transfection. (B) Transfection after 48 hours was validated by immunocytochemistry for depleted NDRG1 protein (brown) in MDA-MB-231 and SUM-159 cells. Blank control represents non-transfected control cells; transfection reagent represents cells in the presence of DharmaFECT 4 transfection reagent only; negative control represents cells transfected with non-targeting control siRNA. (C) Proliferation of SUM-159 (top) and MDA-MB-231 (bottom) cells was determined using the SRB assay at 120 and 72 hours, respectively, post-transfection with *NDRG1* siRNA or non-targeting control siRNA. Experiments were performed in triplicate, and data were normalised to control (untreated) cells; statistical significance was computed using 1-way ANOVA. (D) Migration rate of SUM-159 (top) and MDA-MB-231 (bottom) cells was determined by wound healing assay 48 hours after transfection. Relative migration was determined after 24 hours. Data were normalised to control cells; statistical significance was computed using 1-way ANOVA. ANOVA indicates analysis of variance; SRB, sulforhodamine B.

Comprehensive survival analysis of *NDRG1* in the TCGA RNAseq dataset^12^ that assessed all possible cut-points using the survivALL R package^11^ revealed that 25% (131 of 525) of cut-points were significant (*P* < .05), which is consistent with previous studies of microarray data showing that *NDRG1* expression is positively correlated with invasiveness in breast cancer (Figure 3) and implicated in lipid metabolism.^14,15^ To explore the effect of NDRG1 in the basal subtype of breast cancer, siRNA targeting *NDRG1* and non-targeting siRNA as a negative control were used for transfecting SUM-159 and MDA-MB-231 cells, as well as a mock transfection control (transfection reagent only). Cell lysates were collected at 48, 72, 96, and 120 hours post-transfection. Western blot analysis revealed that maximum *NDRG1* knockdown for SUM-159 cells (84% knockdown) was obtained 48 hours after transfection and that, for MDA-MB-231 cells (89% knockdown), this was at 72 hours post-transfection using 25 nM siRNA (Figure 4A). Immunocytochemistry further confirmed the efficiency of *NDRG1* siRNA transfection compared with the non-targeting siRNA negative control and mock transfection control (Figure 4B). Absence of brown colouration in siRNA-transfected cells confirmed siRNA-mediated knockdown of *NDRG1* in MDA-MB-231 and SUM-159 cells. Proliferation of *NDRG1* siRNA-treated, non-targeting siRNA-treated, mock-transfected, and untreated cells was examined by SRB assay at 48, 72, 96, and 120 hours post-transfection. Optical density values obtained in triplicate at 540 nm were averaged and normalised to day 0 control and untreated control samples at each time point. In SUM-159 cells, a significant reduction in cell proliferation was observed at 120 hours post-transfection (1-way analysis of variance, *P* = .03) (Figure 4C). The MDA-MB-231 cells also showed a decrease in proliferation 72 hours post-transfection, but it was not statistically significant (Figure 4C). To assess the impact of *NDRG1* silencing on the migration of SUM-159 and MDA-MB-231 cells, wound healing assays were performed 48 and 72 hours post-transfection, respectively. Compared with non-targeting siRNA, *NDRG1* siRNA-treated cells did not exhibit any significant changes to cell migration (Figure 4D).

## Discussion

Mitochondria in cancer cells respond to changes in environmental cues by switching between metabolic pathways to help cancer cells survive and proliferate,^2^ but mitochondria-mediated changes in nuclear gene expression are not well studied. It is therefore important to investigate genes whose expression is regulated by mitochondria and how important these are for proliferation of cancer cells. To explore the potential impact of mitochondria on nuclear gene expression, we analysed gene expression data from the cybrid model of triple-negative breast cancer cell lines. Gene expression data from multiple cell line studies and a large patient cohort enabled identification of potential candidate biomarkers (*NDRG1, EXT1, PVT1, F11R, SOX13, ANKRD13B, SCPEP1, NR1D1, CPT1A*). *NDRG1* was observed to be amplified and up-regulated in a large proportion of the basal subtype of triple-negative breast cancer among invasive breast carcinoma patients. NDRG1 has been mostly shown to have a protective function in cancer, although some research suggests that its expression is positively correlated with invasiveness in breast cancer, and it plays an important role in low-density lipoprotein receptor recycling in A431 squamous carcinoma cells.^14,15^ In addition, mutations in *NDRG1* are the cause of Charcot-Marie-Tooth disease type 4D, which is a demyelinating form of Charcot-Marie-Tooth disease that affects the peripheral nervous system.^16^ These studies suggest a possible association of NDRG1 with lipid trafficking and growth in the basal subtype of breast cancer.

Previous work by Park et al and Kaipparettu et al focussed on Src gene signature which is mitochondria regulated and drives malignancy in breast cancer cybrids. However, this is the first time that *NDRG1* silencing has been shown to affect the proliferation of triple-negative breast cancer cells, although it had no effect on cell migration. These results call for a detailed analysis of the molecular mechanism by which mitochondria in metastatic breast cells cause up-regulation of *NDRG1* expression and how NDRG1 regulates the proliferation of SUM-159 cells. One probable explanation for the significant decrease in proliferation in cybrids with benign mitochondria against a metastatic cell nuclear background could be that, in metastatic breast cancer cells, mitochondria-mediated up-regulation of *NDRG1* expression enhances lipid uptake by SUM-159 cells, which may promote cell proliferation in these lipid-dependent cell lines. When a cybrid model is prepared with this metastatic cell line as nuclear background and a benign cell line as mitochondrial donor, *NDRG1* expression is significantly down-regulated, which results in reduced cell proliferation. In contrast, *NDRG1* silencing did not have any significant impact on proliferation and migration of MDA-MB-231 cells. The reduction in proliferation of SUM-159 cells was observed 120 hours post-transfection. At this time point, knockdown of *NDRG1* expression in SUM-159 cells (73% knockdown) was more complete than in MDA-MB-231 cells (58% knockdown). Therefore, more stable silencing of *NDRG1* in MDA-MB-231 cells may enable better evaluation of its impact on proliferation. In a previous study, silencing NDRG1 was found to reduce cell proliferation rates, causing lipid metabolism dysfunction including increased fatty acid incorporation into neutral lipids and lipid droplets. Analysis of public gene expression data also suggests that *EXT1* and *PVT1* may be promising gene targets. These are all present in cytoband 8q24, which is frequently amplified in breast cancer and also in the SUM-159 cell line.^17^ Thus, it would be interesting to evaluate how these genes together affect prognosis in triple-negative breast cancer. We acknowledge the limited nature of this study, but feel that it provides useful supportive data for understanding the role of NDRG1 in breast cancer. In future, it would be of interest to study NDRG1 in the context of other subtypes beyond triple-negative breast cancers.

## Conclusions

This study demonstrates the use of gene expression data to study the impact of mitochondria in proliferation and migration of triple-negative breast cancer cells. Analysis of triple-negative cell lines and cybrids identified potential mitochondria-regulated gene candidates that were evaluated in clinical datasets. *NDRG1* was found to be the most up-regulated gene in cybrids with cancer cell–derived mitochondria, and it is amplified and up-regulated in a large fraction of patients with the basal subtype of invasive breast carcinoma.^17^ Silencing *NDRG1* significantly reduced proliferation, but has no effect on migration, of SUM-159 cells. Further investigations on how NDRG1 affects low-density lipoprotein uptake in triple-negative breast cancer may provide new therapeutic insights in the treatment of breast cancer.
